# Multimodal imaging platform for rapid non-destructive evaluation of pancreatic puncture specimens

**DOI:** 10.1117/1.JBO.31.7.076502

**Published:** 2026-07-06

**Authors:** Jianan Zhang, Qing Zhang, Jiansheng Wang, Chunhua Zhou, Lili Gao, Zhen Sun, Yan Wang, Duowu Zou, Qingli Li

**Affiliations:** a East China Normal University, Shanghai Key Laboratory of Multidimensional Information Processing, Shanghai, China; b Ruijin Hospital, Shanghai Jiao Tong University, School of Medicine, Department of Gastroenterology, Shanghai, China; c Ruijin Hospital, Shanghai Jiao Tong University, School of Medicine, Department of Pathology, Shanghai, China; d Four Districts for Convalescence of Air Force Hangzhou Special Service Recuperation Center, Jiangsu, China

**Keywords:** hyperspectral imaging, support vector machine, endoscopic ultrasound-guided fine needle biopsy, macroscopic visible core adequacy evaluation, rapid on-site evaluation, macroscopic on-site evaluation

## Abstract

**Significance:**

Destructive staining and slow subjective visual inspection remain major limitations in rapid evaluation of biopsy specimens during endoscopic ultrasound-guided fine needle biopsy. Therefore, there is a strong need for a label-free optical imaging approach that enables quantitative specimen assessment while preserving specimen integrity.

**Aim:**

A multimodal imaging system integrating hyperspectral and color imaging was developed to acquire images of biopsy specimens without staining or sample preparation.

**Approach:**

A hyperspectral on-site evaluation workflow was proposed, integrating hyperspectral imaging and color imaging within a modular platform. Hyperspectral data were analyzed using a support-vector-machine-based segmentation framework for specimen assessment with color images providing reference annotations. No washing or staining is required.

**Results:**

Validation on 220 pancreatic biopsy specimens demonstrates accurate non-destructive macroscopic visible core evaluation, achieving an F1-score of 94.7%. The proposed workflow improves analysis efficiency by 93% compared with conventional rapid on-site evaluation.

**Conclusions:**

We demonstrate that integrating hyperspectral imaging with computational analysis enables rapid, quantitative, and label-free specimen evaluation, highlighting the potential of biomedical optical systems for intra-procedural specimen assessment.

## 1 Introduction

Pancreatic diseases, especially pancreatic cancer, have always been a major challenge in medical field due to its high lethality and complex diagnostic process.^1^ Conventional non-invasive imaging modalities (e.g., computed tomography or magnetic resonance imaging) are insufficient for the definitive diagnosis of pancreatic cancer. The gold standard for a definitive diagnosis relies strictly on extracting actual pancreatic tissue for histopathological staining and analysis. Clinically, accessing the pancreas requires a minimally invasive procedure known as endoscopic ultrasound-guided fine needle biopsy (EUS-FNB), where a needle is passed through the stomach wall into the pancreas to extract tissue. However, because pancreatic tumors are highly fibrotic, the extracted specimen is rarely pure; it is typically a chaotic mixture of target tissue [macroscopic visible cores (MVCs)], large amounts of blood, and mucin.^2^^–^^4^ For clinicians, the immediate challenge during the procedure is determining whether enough target MVC tissue has been successfully extracted through EUS-FNB. If the sample is inadequate, the patient must undergo repeated punctures. Therefore, there is a critical engineering and clinical need for a rapid, non-destructive analytical tool that can quantify the target tissue content to guide surgical decision-making. MVCs refer to the intact, whitish or yellowish tissue cylinders acquired during the EUS-FNB procedure. Because pancreatic tumors are characteristically dense and highly desmoplastic, obtaining an adequate amount of preserved tissue architecture is a critical hallmark of a successful puncture. Acquiring an adequate proportion of MVCs is a fundamental prerequisite for successful downstream pathological evaluation. Insufficient tissue material severely limits the preparation of high-quality histological slides, fundamentally impeding the pathologist’s ability to definitively diagnose the specific condition and staging of the pancreatic cancer. Clinical studies have consistently demonstrated that the presence and proportional area of these macroscopic white cores are directly correlated with diagnostic adequacy, ensuring sufficient tumor and stromal tissue for definitive histopathological and molecular analysis.^5^^,^^6^ However, the specimen obtained by EUS-FNB usually contains MVCs, blood, and some other exudates, which makes it difficult for clinicians to determine whether the MVCs meet the requirements by manual observation. Rapid on-site evaluation (ROSE)^7^^–^^9^ and macroscopic on-site evaluation (MOSE)^10^^,^^11^ are the two recommended protocols during EUS-FNB to assess MVC adequacy.

According to clinical guidelines issued by major medical authorities, such as the European Society of Gastrointestinal Endoscopy (ESGE)^12^ and the American Society for Gastrointestinal Endoscopy (ASGE),^13^ ROSE is highly recommended during EUS-FNB to assess sample adequacy and maximize diagnostic yield.^14^ However, despite this institutional endorsement, its implementation is time-consuming and heavily depends on the immediate availability of experienced pathologists, largely restricting its universal application.^9^ It begins with a rapid staining of the cell smear from the pancreatic puncture specimen. The pathologist then makes a diagnosis by observing the stained cell smear through a microscope. Finally, the clinician decides whether an adequate MVC has been obtained from EUS-FNB based on the pathological diagnosis. In addition, ROSE is a destructive evaluation method that requires removing part of the specimens for staining analysis. The specimens used cannot be used for further diagnosis. This sampling-based approach inevitably reduces the remaining tissue available for subsequent pathological analysis.

Due to the shortcomings of ROSE, MOSE^10^^,^^11^ has been increasingly adopted as a practical alternative protocol during EUS-FNB when ROSE is unavailable according to the ESGE and ASGE guidelines. MOSE involves the immediate visual inspection of unstained specimens by clinicians. Studies by Iwashita et al.^5^ and Chong et al.^6^ demonstrated that visually estimating MVC length can reliably indicate diagnostic adequacy, thereby requiring fewer puncture attempts. It is better for the patient’s health. However, MOSE relies entirely on human observation. This subjective eyeballing lacks standardized quantitative criteria, making it difficult for clinicians to accurately and consistently evaluate the true proportion of MVCs amidst blood.

Recent advancements have introduced optical instruments to assist specimen evaluation. Masutani et al.^15^ and Okuwaki et al.^16^ utilized stereomicroscopes to observe MVCs, while Amendoeira et al.^17^ explored fluorescence confocal microscopy. Furthermore, Matsumoto et al.^18^ utilized hemoglobin’s absorption spectrum at 605 nm to visually separate MVCs from blood. Despite these optical innovations, these methods primarily assist manual observation. They can determine the presence of MVCs but lack the computational ability to automatically analyze the proportion of MVC content, leaving the final assessment to the pathologist’s judgment. The requirements of clinicians and pathologists are not always consistent. Pathologists need to analyze processed samples for a long time to obtain results, while clinicians need a fast and automated diagnostic method so that it can be easily embedded into the fast-paced surgical process. Given the limitations of the above methods, a preferable strategy is to enable a fast and non-destructive quantitative method without the involvement of pathologists.

Hyperspectral imaging (HSI) presents a powerful optical alternative^19^^–^^23^. Unlike conventional color [red, green, and blue (RGB)] imaging that captures light in only three broad bands (red, green, and blue), HSI separates broadband light passing through the sample into distinct narrow-band wavelengths using a tunable filter, allowing a monochrome camera to capture a sequence of single-band grayscale images that are subsequently stacked to form a three-dimensional (3D) spatial–spectral data cube. In the context of pancreatic biopsy evaluation, this dense spectral information is highly advantageous. Raman imaging provides highly specific molecular vibrational information and is valuable for label-free tissue analysis. However, its relatively weak signal, longer acquisition time, and more complex optical configuration may limit its practicality for rapid assessment. The proposed HSI approach enables rapid acquisition of wavelength-dependent absorption information from unstained specimens, making it more suitable for macroscopic puncture specimen evaluation. When MVCs are heavily coated in blood and exudates, they often appear visually indistinguishable from the surrounding materials to the naked eye or a standard RGB camera. However, because different biomolecules have distinct light transmission properties, they have unique spectral signatures. Accurately identifying subtle spectral features through machine learning algorithms eliminates the need for additional sample processing.

This is fundamentally due to the distinct biochemical compositions of the sample components, which dictate specific light absorption and transmission properties.^23^^,^^24^ For example, blood is rich in hemoglobin, which exhibits multiple characteristic absorption-related spectral bands in the visible range of 500 to 700 nm, including prominent absorption features around ∼540 and 640 nm. Conversely, the MVCs, which are composed of desmoplastic stroma and cellular structures, lack these specific absorption signatures and interact with light primarily through different scattering mechanisms.^19^^,^^24^ Although conventional RGB cameras average out these subtle variations over broad color bands, HSI’s narrow wavelength resolution captures these endogenous biochemical fingerprints, enabling the identification of specific tissue components based on their spectroscopic properties. HSI was integrated into an endoscopic system with a liquid crystal tunable filter,^25^ enabling label-free, real-time detection of pancreatic tumors in mouse models. Their system demonstrated exceptional tissue discrimination capability and robustness against biological interference (e.g., blood suppression). A deep learning framework was established based on HSI data of Hematoxylin and Eosin-stained samples, improving the diagnostic accuracy of pancreatic ductal adenocarcinoma to 92.04%, which outperforms the color image-based model.^26^ Researchers achieve classification of pancreatic neuroendocrine tumor cells and benign pancreatic cells in liquid-based cytology specimens by a combination of hyperspectral imaging and deep learning algorithms.^27^ Results on hyperspectral images provide superior classification performance compared with conventional color images. A diagnostic method based on hyperspectral images using partial least squares and discriminant analysis^28^ was proposed to help achieve specimen classification in unstained pancreatic cancer tissue sections. This indicates that no staining of the specimen is required for the use of hyperspectral imaging. Although HSI enables blood interference suppression and quick tissue quantification through rich spectral information, its data format (3D spectral cube) is difficult to be directly interpreted by pathologists. Color imaging can serve dual roles as a clinically interpretable interface and a morphological annotation aid. Color images are a common image modality in conventional diagnosis, which provide intuitive morphological references, allowing pathologists to rapidly localize regions of interest and validate the diagnostic rationality.

Conventional methods using a single modality for assessing pancreatic specimen adequacy face a trilemma among speed, non-destructiveness, and objectivity. Recent advances in multimodal imaging platforms have demonstrated potential for pancreatic diagnostics by synergizing complementary data sources.^29^^–^^31^ The integration of different data source, often with artificial intelligence, is transforming pancreatic cancer diagnosis by providing more precise, real-time insights into disease progression and treatment effectiveness.^32^

Motivated by these observations, we propose hyperspectral on-site evaluation (HOSE), a rapid and non-destructive workflow for quantitatively assessing MVC adequacy. The workflow consists of two key components. First, a multimodal imaging platform integrates HSI and color imaging to acquire paired images of pancreatic biopsy specimens. Second, a data analysis module enables quantitative assessment by segmenting hyperspectral images. The segmentation is performed using a support vector machine (SVM) trained on clinician-annotated MVC regions from the color images. Our contributions are summarized as follows:

• We propose a rapid, non-destructive, and quantitative workflow, named HOSE, which streamlines MVC assessment from specimen acquisition to clinical decision-making.
• We propose a unique multimodal imaging platform based on a cage structure, with hyperspectral and color imaging for pancreatic puncture specimens.
• We evaluate the proposed HOSE workflow on a self-collected dataset of 220 pancreatic puncture specimens. The results demonstrate that HOSE achieves high performance in terms of time efficiency, quantitative capability, and non-destructiveness, with a diagnostic F1-score of 94.7% and an average diagnosis time of only 1.5 min.

## 2 Materials and Methods

### 2.1 Overall Architecture of the HOSE Workflow

**Fig. 1:**
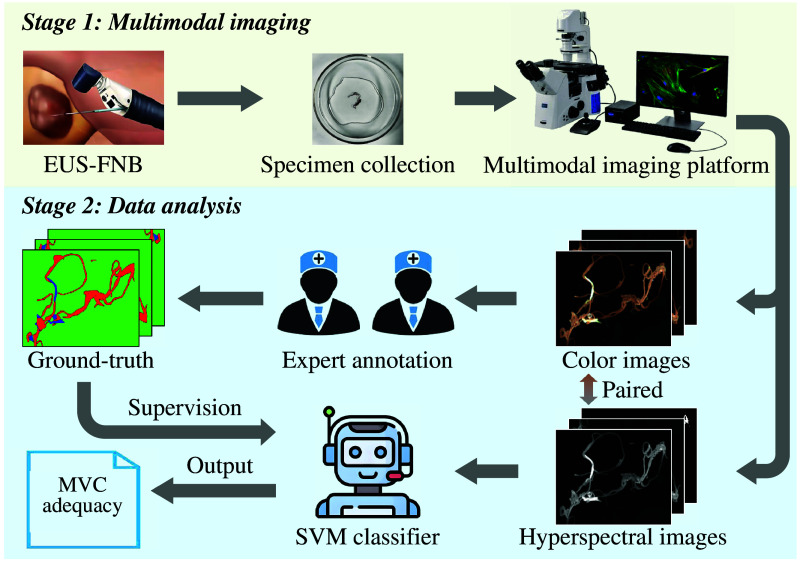
Proposed hyperspectral on-site evaluation workflow.

Figure 1 illustrates the HOSE workflow designed for rapid, non-destructive, and quantitative assessment of MVC in pancreatic biopsy specimens.

In stage 1, after completing the puncture, the specimen in the needle is placed in a Petri dish on the imaging platform to minimize tissue fragmentation caused by manual handling. Paired hyperspectral and color images are then acquired using a multimodal imaging platform under standardized illumination conditions at 0.5× magnification, preserving tissue integrity for downstream pathological analysis.

In stage 2, to eliminate any potential cross-modality pixel misalignment errors during model training, the ground truth annotations for MVC and blood are delineated by experienced doctors independently on both the color images and the hyperspectral images. Consequently, the pixel-level masks annotated directly on the spatial coordinates of the hyperspectral images serve as the exact ground-truth for training the SVM classifier. During clinical application, new hyperspectral images are processed by the trained SVM to generate MVC segmentation maps, enabling quantitative evaluation of MVC content based on the ratio of MVC area to total tissue area. This process yields a continuous adequacy score ranging from 0% to 100%. If the score falls below 40%, the system automatically triggers audiovisual alerts to prompt timely re-biopsy decisions.

All imaging and analysis results are stored in a local database to ensure traceability and support multidisciplinary clinical consultations.

### 2.2 Multimodal Imaging Platform

**Fig. 2:**
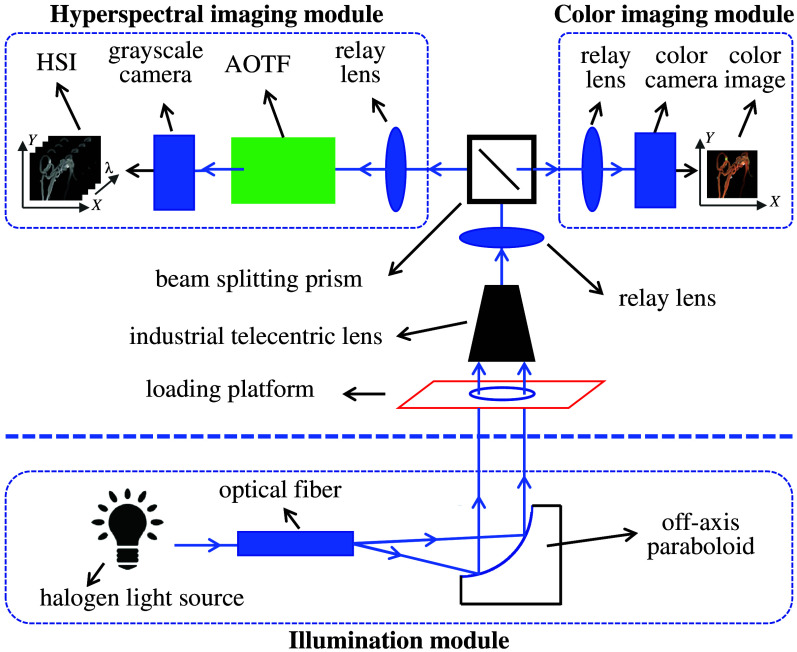
Schematic diagram of the structure and optical path of the multimodal imaging platform.

To enable rapid, accurate, and non-destructive evaluation of MVC adequacy, we develop a unified dual-channel imaging platform that simultaneously acquires color images and hyperspectral data, as shown in Fig. 2. All components are rigidly installed within a modular cage frame, which has mechanical stability and optical path multiplexing architecture.

The illumination module consists of a broadband halogen lamp and an off-axis parabolic mirror. A stabilized halogen lamp (150 W) supplies a smooth and continuous spectrum that satisfies the requirements for spectral continuity and intensity of the light source in hyperspectral imaging. The lamp output is coupled into an optical fiber, which transports the light to the off-axis parabolic mirror while isolating heat and electromagnetic noise. At the fiber terminus, a conical divergent beam is incident on an off-axis parabolic mirror, which collimates the light into a parallel beam. The resulting collimated light uniformly illuminates the sample plane. The loading platform is designed to accommodate Petri dishes with specimens. Specimens are illuminated by the illumination module and imaged through the industrial telecentric lens.

To extend the optical path of the imaging system and improve imaging stability, we have placed relay lenses between different modules. The color imaging module comprises a relay lens and a color camera positioned on one arm of the beam-splitting prism. Incoming light from the industrial telecentric lens is directed through the beam splitter into the color imaging module. The hyperspectral imaging module consists of a relay lens, a commercial acousto-optic tunable filter (AOTF), and a grayscale camera. The commercial AOTF operates as a rapid, electronically controlled optical bandpass filter. By modulating the applied radio-frequency signals, the AOTF selectively diffracts specific narrow spectral bands within the 450- to 750-nm range from the incident broadband light. AOTF provides selective spectral separation over 450 to 750 nm. After the beam splitter directs the transmitted light into the hyperspectral imaging module, the imaging lens focuses the scene onto the AOTF, which electronically selects narrow spectral bands with nanometer precision. The filtered single-band light is then re-imaged onto the grayscale detector, capturing a single-frame image in the designated wavelength. By controlling AOTF to filter the incident light, the camera can obtain grayscale images of specimens at different wavelengths.

Because the color camera and the grayscale camera have different physical dimensions and are located on different arms of the beam splitter, they exhibit slight differences in magnification and field of view (FOV). For the purpose of overall system spatial calibration and paired visual comparison, we performed a spatial alignment step. Specifically, the acquired high-resolution color images are subjected to a scaling and cropping operation to exactly match the magnification and FOV of the hyperspectral images.

### 2.3 Data Analysis

The acquired hyperspectral data consist of 40 discrete spectral bands spanning from 450 to 750 nm. To ensure fidelity and computational efficiency, the data cube first undergoes a preprocessing workflow, including calibration and spectral smoothing.

• Transmittance calibration: Converts raw digital numbers to transmittance values
  $$T(\lambda )=\frac{{\mathrm{DN}}_{\text{specimen}}(\lambda )-{\mathrm{DN}}_{\text{dark}}(\lambda )}{{\mathrm{DN}}_{\text{blank}}(\lambda )-{\mathrm{DN}}_{\text{dark}}(\lambda )},$$ (1)
  where T(λ) is the calibrated transmittance at wavelength λ, DN denotes digital number, and subscripts indicate measurement conditions at wavelength λ.
• Spectral smoothing: The Savitzky–Golay filter performs a local polynomial regression of degree k on a series of values in a moving window of size n=2m+1. The filtered value t^0 is determined by
  t^0=e1T(XTX)−1XTT, (2)
  where $T=[t_{-m},\ldots ,t_{0},\ldots ,t_{m}{]}^{T}$ represents the observation vector of raw transmittance values within the window, X is the (2m+1)×(k+1) design matrix with elements $X_{i,j}=i^{j}$ (for −m≤i≤m and 0≤j≤k), and $e_{1}^{T}=[1,0,\ldots ,0]$ is a unit selection vector used to extract the zeroth-order coefficient of the fitted polynomial.

Following preprocessing, an SVM-based pixel-level segmentation algorithm is employed to accurately segment MVCs from the surrounding non-core regions within the specimen. Each pixel of the acquired HSI data is used as an input feature for training an SVM classifier. The MVC adequacy score for each biopsy specimen is computed as the proportion of pixels classified as MVC, providing a continuous score ranging from 0% to 100%.

## 3 Results

### 3.1 Experimental Design

#### 3.1.1 Dataset description

In this study, 220 pancreatic biopsy specimens were obtained from 44 unique patients at Ruijin Hospital between 2023 and 2024 via EUS-FNB using 22-gauge needles. To establish a robust dataset and validate our proposed label-free workflow, we separated the data acquisition into two steps. First, freshly acquired, unstained specimens were placed in a Petri dish, and macroscopic hyperspectral and color images (0.5×) were immediately captured using our multimodal imaging platform. Second, to establish the definitive ground truth, these same specimens underwent routine pathological processing into H&E-stained slides, which were then scanned at 20× magnification using a digital slide scanner.

To ensure reliable ground truth without subjective bias, a dual-level annotation strategy was employed. At the specimen level, senior pathologists evaluated the microscopic H&E images to classify each specimen as MVC adequate or inadequate. At the pixel level, clinicians utilized the paired high-resolution macroscopic color images as precise spatial references to annotate MVC, blood, and background regions on the HSI data.

To prevent data leakage during model training and evaluation, the 220 specimens were divided into a training set (176 specimens) and a testing set (44 specimens) strictly using a patient-level split. This ensures that all specimens from any single patient were exclusively assigned to either the training set or the testing set. By correlating the macroscopic MVC ratios (calculated from pixel-level annotations) with the pathologists’ H&E adequacy labels within the training set, a critical threshold of 40% was statistically established (i.e., an MVC ratio≥40% is quantitatively considered adequate).

#### 3.1.2 Imaging platform specifications

The imaging platform’s performance is quantified through the following critical parameters:

• Spectral resolution (Δλ): Determines the minimum distinguishable wavelength interval
  $$\Delta \lambda =\frac{\lambda_{\max}-\lambda_{\min}}{N_{\text{bands}}-1},$$ (3)
  where $\lambda_{\max}=750\;\mathrm{nm}$ and $\lambda_{\min}=450\;\mathrm{nm}$ define the operational spectral range, and $N_{\text{bands}}=40$ denotes the number of spectral bands.
• Spatial resolution ($R_{\text{spatial}}$): We use an internationally recognized standard test tool (the USAF-1951 resolution test board) to calculate the spatial resolution performance of the imaging component. The conversion of spatial resolution $R_{\text{spatial}}$ of the USAF-1951 resolution test board is as follows:
  $$R_{\text{spatial}}=2^{\alpha +(\beta -1)/6},$$ (4)
  where α represents the group number of the USAF-1951 resolution test board, and β represents the target line number within the group.

#### 3.1.3 Analysis algorithm configuration

The SVM classifier is implemented using Scikit-learn (v1.5.0). Specifically, a radial basis function kernel is employed to handle nonlinear feature distributions. The regularization parameter C is set to 10.0 to balance model complexity and classification accuracy, and the kernel coefficient (gamma) is fixed at 0.001 to control the influence range of individual samples in the high-dimensional feature space. To address class imbalance, class weights are adjusted using an inverse frequency strategy. All experiments are conducted on a workstation running the Windows 10 operating system, with implementation carried out in Python version 3.6.

### 3.2 Results

#### 3.2.1 System characterization

**Fig. 3:**
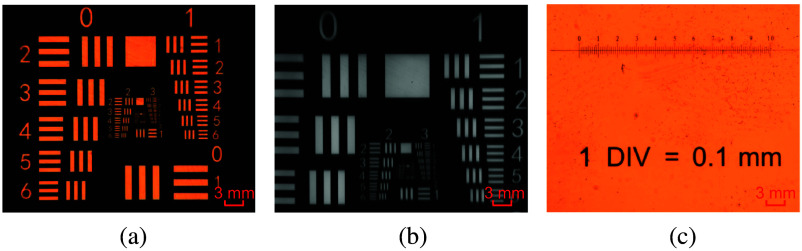
Characterizations of our imaging platform. (a) Color image acquired using a USAF-1951 resolution test board. (b) Corresponding grayscale image at 616 nm. (c) Color image of 0.1-mm-divided ruler to calibrate the field of view range.

As shown in Fig. 3, imaging performance across different modalities is assessed using the USAF-1951 resolution test board, a calibrated ruler, and a grid standard board. Based on Eq. (4), the system’s macroscale spatial resolution is 8.98  lp/mm. According to Eq. (3), the spectral resolution of the system is 7.69 nm. These results demonstrate consistent contrast, sharpness, and negligible distortion, validating the system’s superior spatial resolution and suitability for specimen acquisition.

#### 3.2.2 Diagnostic performance of HOSE

We compare our method with two competitive approaches, namely, ROSE and MOSE, as summarized in Table 1. Due to the fact that ROSE and MOSE can only qualitatively evaluate whether the specimen is qualified and cannot accurately segment the MVC region, we compare the effectiveness of these methods in evaluating the MVC content using classification metrics precision, recall, and F1-score. Specimens with sufficient MVC (≥40%) are one type, while specimens with insufficient MVC (<40%) are another type. Precision reflects the proportion of correctly identified adequate specimens among all those predicted as adequate, while recall measures the ability to correctly retrieve all truly adequate specimens. The F1-score, as the harmonic mean of precision and recall, provides a balanced measure of diagnostic accuracy under imbalanced data conditions. Additional time and wastage indicators have been added. The time index aims to evaluate the average specimen processing time from specimen acquisition to providing diagnostic results. To ensure a fair and objective comparison, the processing time for all methods is strictly evaluated as an end-to-end procedure. The timing protocol starts the moment the extracted specimen is deposited into the Petri (or onto a slide) and concludes when the final adequacy diagnostic result is provided. The wastage index aims to evaluate the average proportion of specimens consumed during the diagnostic process.

**Table 1:** Comparative performance evaluation of different methods.

Method	Precision (%)	Recall (%)	F1-score (%)	Time (min)	Wastage (%)
ROSE	100.0	100.0	100.0	22.3	15.0
MOSE	100.0	70.0	82.4	14.5	5.0
**HOSE (ours)**	**100.0**	**90.0**	**94.7**	**1.5**	**0.0**

Note: bold values indicate the results of the proposed HOSE workflow.

All three methods achieve perfect precision (100.0%), demonstrating their ability to accurately identify specimens with sufficient MVC. Among them, ROSE achieves the highest recall and F1-score, while HOSE shows slightly lower values than ROSE but remains markedly superior to MOSE. HOSE substantially outperforms both ROSE and MOSE in processing time, requiring only 1.5 min compared with 14.5 min for MOSE and 22.3 min for ROSE, owing to its automated imaging and data analysis pipeline. The time for ROSE (average 22.3 min) encompasses manual smear preparation, rapid chemical staining, and detailed microscopic examination by a pathologist. The time for MOSE (average 14.5 min) involves careful physical sorting of the specimen using forceps and subsequent subjective visual inspection by the clinician. Conversely, HOSE computes the result continuously from the moment the sample is placed in the Petri. HOSE breaks down into ∼45  s for data scanning and 45 s for data processing and output results. By minimizing processing time, HOSE significantly reduces surgical duration, which is critical for patient outcomes. Furthermore, HOSE achieves a specimen wastage rate of 0%, compared with 5% for MOSE and 15% for ROSE, fully preserving specimen integrity for subsequent histological validation.

**Fig. 4:**
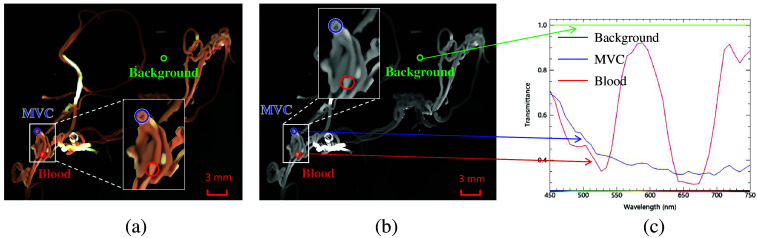
Pancreatic puncture macroscopic specimen imaging results. (a) Color image. (b) Hyperspectral single-band image in the 640-nm band. The specimens at the blue point are MVCs, and the specimens at the red point are blood. The green point position is background. (c) Comparison of spectral profiles of three regions of the specimen.

In the process of diagnosing the adequacy of MVCs, we focus on the differences in spectral curves of different organizational locations. Figure 4 demonstrates the corresponding spectral profiles of the hyperspectral images obtained from our system. Figures 4(a) and 4(b) are a set of color images and hyperspectral single-band images. Figure 4(c) shows the spectral curve of the hyperspectral image. The positions of the corresponding pixels are marked with dots in Fig. 4(c), where the green curve corresponds to the background, the blue curve corresponds to MVC, and the red curve corresponds to blood. In the color images of the pancreatic biopsy specimen shown in Fig. 4(a), the MVC regions covered by blood are difficult to visually identify. However, the hyperspectral data show characteristic decreases in blood transmittance around ∼540 and 640 nm, which are consistent with previously reported hemoglobin-related spectral features. In addition, MVC exhibits pronounced spectral differences from blood around ∼540, 580, and 640 nm. This spectral distinction is the principal factor enabling the precise and successful quantification of MVC content using hyperspectral imaging instead of standard color imaging. For the collected specimens, HSI provides significantly richer information than conventional color images by capturing fine spectral variations across different wavelengths. These subtle differences, which are imperceptible in RGB images, enable a more accurate characterization of tissue composition and pathological features. Representative grayscale images across all 40 hyperspectral bands are provided in Fig. S1 in the Supplementary Material, showing the wavelength-dependent intensity variations captured by our system.

### 3.3 Ablation Study

Table 2 presents the ablation study comparing the classification performance between color images and hyperspectral images using three different classifiers. Across all classifiers, hyperspectral images consistently outperforms color images in terms of precision, recall, and F1-score, demonstrating the advantage of leveraging richer spectral information for more accurate classification. Among the tested classifiers, SVM achieves the best overall performance with hyperspectral images, obtaining an average performance of 94.9%, which substantially surpasses the corresponding results achieved with color images (68.51%). These results emphasize that integrating hyperspectral imaging with SVM can provide the most robust and accurate classification performance, demonstrating its enormous potential in improving diagnostic reliability in clinical workflows.

**Table 2:** Ablation on the image modality and the classifier. The average value is the mean of precision, recall, and F1-score.

Data Modality	Classifier	Precision (%)	Recall (%)	F1-score (%)	Average (%)
Color image	kNN	60.0	40.0	48.0	49.3
	Random forest	70.0	50.0	58.3	59.4
	SVM	77.8	60.0	67.7	68.5
Hyperspectral image	kNN	85.7	80.0	82.8	82.8
	Random forest	92.3	90.0	91.1	91.1
	**SVM**	**100.0**	**90.0**	**94.7**	**94.9**

Note: bold values indicate the results of the proposed HOSE workflow.

**Fig. 5:**
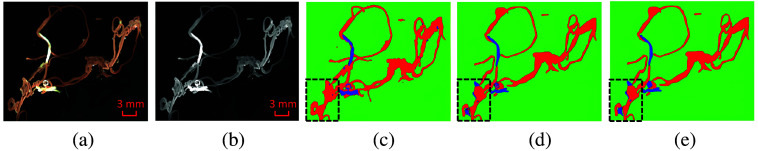
Segmentation results of pancreatic puncture specimens utilizing SVM. (a) Color image. (b) Hyperspectral single-band image at 640 nm. (c) Segmentation result based on the color image. (d) Segmentation result based on the hyperspectral image. (e) Hyperspectral image ground-truth annotation (red for blood, blue for MVC, and green for background).

Figures 5(c) and 5(d) show the results of our algorithm. Comparison with ground-truth shows that the results based only on color image processing miss a portion of valid MVC, while the results based on hyperspectral data processing effectively find the MVC covered by blood. This segmentation has been professionally validated by experienced pathologists, confirming that the hyperspectral images captured by our system are rich in diagnostic information. Figure S2 in the Supplementary Material shows another representative specimen with comparisons between the color image and selected hyperspectral band images. The MVC and blood regions that are visually similar in the color image can be more clearly distinguished at specific wavelengths. By analyzing this data, pathologists can accurately evaluate the tissue strip content and assess the adequacy of the puncture, thereby enhancing the precision and efficiency of puncture procedures. To identify which bands are the most informative in hyperspectral data, we performed an image-entropy-based band importance assessment, as shown in Fig. S3 in the Supplementary Material. Briefly, low-variance and highly redundant bands were removed through variance filtering, correlation analysis, hierarchical clustering, and K-means grouping. Within each group, the band with the highest image entropy was selected as the representative wavelength. This strategy is consistent with recent medical hyperspectral image studies.^33^ The analysis identified 526.9, 534.6, 565.4, 580.8, and 626.9 nm as the most informative bands, which are mainly located within the regions associated with characteristic hemoglobin absorption.

## 4 Conclusion

ROSE and MOSE are the current standard workflows for Endoscopic Ultrasound-Guided Fine Needle Aspiration diagnosis, yet both have limitations in primary healthcare settings due to resource constraints and efficiency requirements: (1) destructive real-time assessments, (2) time-intensive workflows prolonging surgical procedure, and (3) subjective quantification relying on clinicians’ experience. To address these issues, we develop a rapid and non-destructive workflow for evaluating MVC adequacy, integrating a multimodal imaging platform with an automated data analysis module. By comparing with the standard surgical procedure, our HOSE method provides a practical compromise by maintaining comparable diagnostic accuracy, achieving superior efficiency and zero specimen wastage, and improving recall over MOSE. These characteristics make HOSE a scalable and resource-efficient solution, particularly suitable for enhancing diagnostic capability in primary and community hospitals.

Despite promising initial results, this study is limited by the relatively small size of dataset, which may have constrained the model’s ability to learn comprehensive feature representations and its diagnostic robustness. In future work, we will expand our data collection efforts to assemble a larger and more diverse set of hyperspectral images, thereby enabling more thorough training and validation. Although the current 40-band spectral sweep successfully captures comprehensive biochemical signatures, this high-dimensional data acquisition inevitably introduces spectral redundancy. In future work, we will conduct feature importance and band entropy analyses to isolate the most discriminative wavelengths. By optimizing the system to acquire only a few critical bands, we aim to significantly reduce data redundancy and drastically accelerate the optical acquisition process, thereby further enhancing the system’s efficiency for real-time clinical deployment. Furthermore, we plan to explore the integration of large pre-trained models and transfer learning techniques to enhance the generalizability of our diagnostic framework across varied tissue types and imaging conditions. Together, these efforts are expected to substantially strengthen the model’s performance and clinical applicability.

## Supplementary Material

10.1117/1.JBO.31.7.076502.s01

## Data Availability

The code and data are available on request.

## Patents

The proposed HOSE workflow and the multimodal imaging platform are covered by a granted Chinese patent entitled “Pathological Analysis System and Method for Puncture Specimens Based on Hyperspectral Imaging Technology” (Patent No. ZL 2022 1 0863506.X).
