# Supplementary material for: Multimodal imaging platform for rapid non-destructive evaluation of pancreatic puncture specimens
Source: J Biomed Opt. 2026 Jul 6;31(7):076502. doi: 10.1117/1.JBO.31.7.076502 (PMC13336349; doi:10.1117/1.JBO.31.7.076502)
Supplement: Supplementary file 1 [file JBO_031_076502_SD001.pdf]

Supplementary Figures

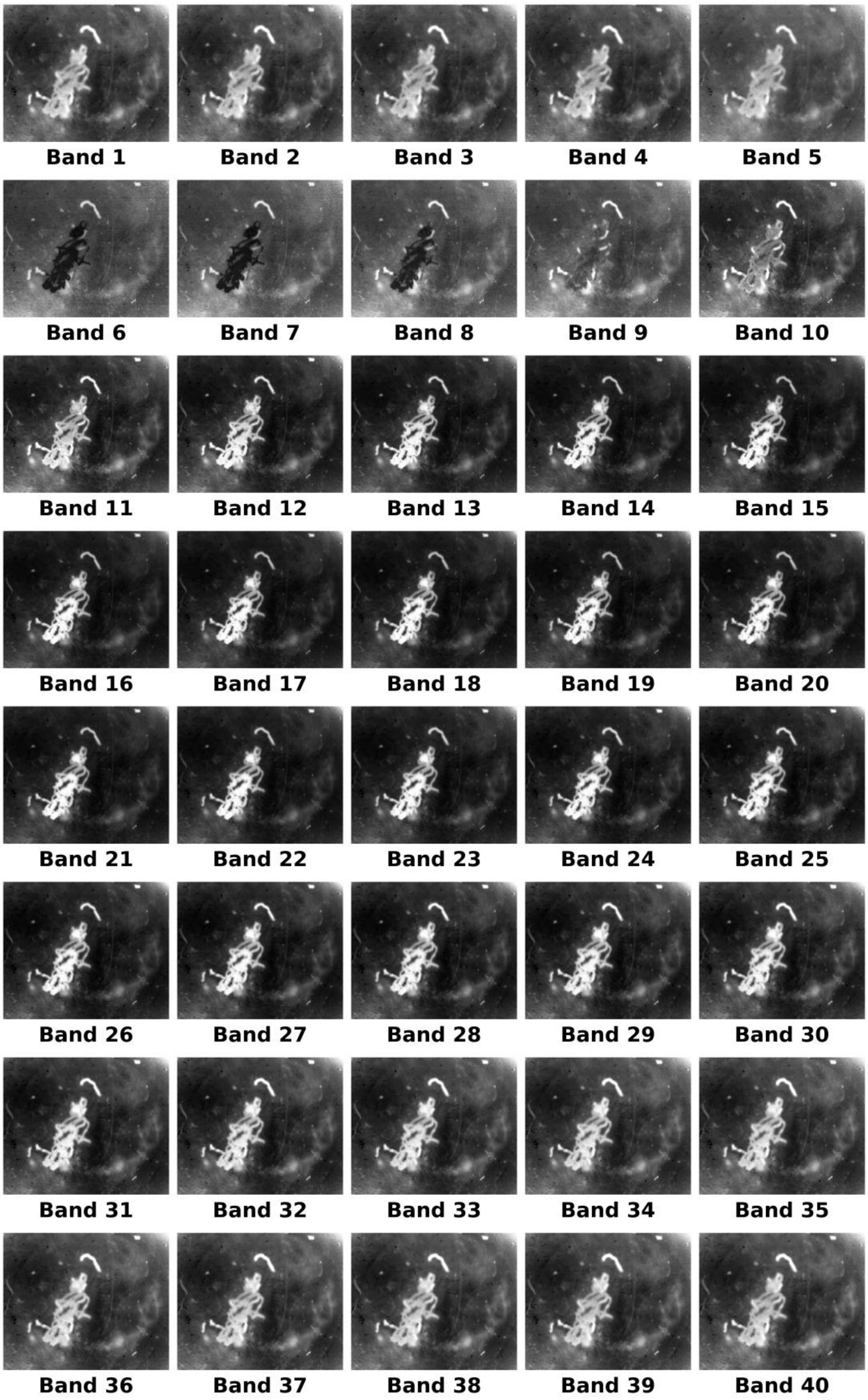

Supplementary Fig S1 One example of the HSI at all bands.

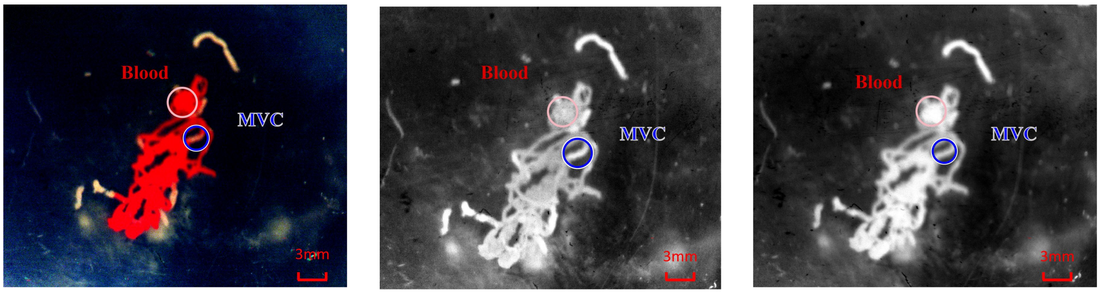

(a) Color image (b) Hyperspectral image band 12 (c) Hyperspectral image band 28

Supplementary Fig S2 Comparison between color image and some important bands. (a) Color image. (b) Hyperspectral image band 12. (c) Hyperspectral image band 28.

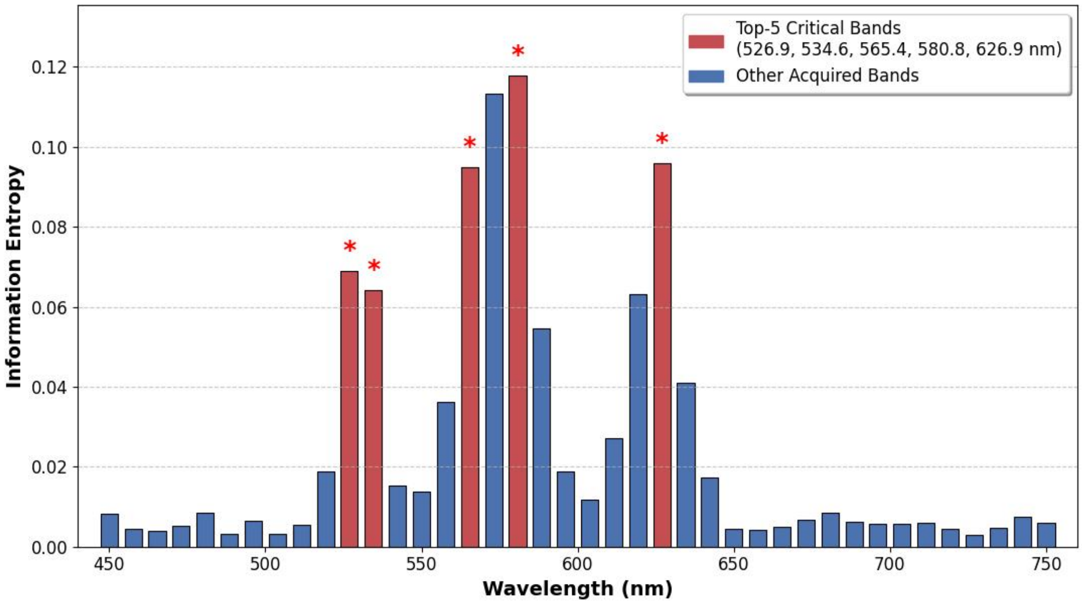

Supplementary Fig S3 Analysis of Band Importance.
